# Powders with zero Ohmic resistance

**DOI:** 10.1038/s41598-022-22452-1

**Published:** 2022-10-19

**Authors:** Vladimir F. Kharlamov

**Affiliations:** Orel State University, Orel, Russia 302026

**Keywords:** Nanoscale materials, Theory and computation

## Abstract

We substantiate the mechanism of electrical conductivity of powder consisting of dielectric nanoparticles with ionized donor centers on the surface and free electrons in the bulk. It is shown that in a powder switched to a closed dc circuit, the electric field strength vanishes due to the polarization of the powder by the source of the electromotive force. The Ohmic loss becomes impossible. The electrons of the polarized powder are in the states that correspond to the minimum of the powder energy and zero Ohmic resistance. The fulfillment of these conditions does not depend on the temperature (*T* = 300–500 K) and the strength of the circuit current. The electric current in the powder is maintained on account of the difference in the electrochemical potentials of electrodes that are in contact with the polarized powder.

## Introduction

Superconductivity of solid-states is explained by mutual attraction between free electrons. This attraction comes into play when the interaction of electrons with the lattice and other electrons is accounted for^1,2^. In the theory of superconductivity, the attraction energy of two electrons is negative, *w*_e_ < 0. The presence of a medium is account for by introducing the dielectric permittivity ε. At ε < 0, we have *w*_e_ = *e*^2^/(ε*r*) < 0. Since the condition ε < 0 holds true, the charge carriers of the system participate in the exchange–correlation interaction. The equations of high-temperature superconductivity theory have been derived that describe the mutual attraction of electrons as caused by the negative static permittivity of a solid ε = ε(0, **k**). For the energy gap and the wave function of the cooper pair, they are homogeneous Eqs. 1, 2.

For media with spatial dispersion, the application of the equations of electrodynamics of continuous media is restricted^2,3^. The effective field acting on charged particles of a disperse system differs from the average macroscopic field. For this reason, the electrical processes in disperse systems have their specific features as contrasted to similar processes occurring in homogenous systems. Studies have been conducted that deal with electron phenomena in solids with a granular structure. For example, compounds based on transition metal oxides are high-temperature superconductors that can, under certain conditions, exist in a state of periodic phase separation into semiconductor and metal phases^4^. Composite and metamaterials with a negative real part of the permittivity ε′ have been fabricated. They are characterized by Ohmic loss and frequency dispersion ∂ε′/∂ω^5–7^.

According to the Kramers–Kronig dispersion relations, for the static permittivity of any substance one of the following conditions is satisfied: ε ≥ 1 or ε ≤ 0^2^. For a long time, the possibility of producing materials with ε ≤ 0 was a controversial issue^1,2^. Their structure and some properties were first substantiated in article^8^. The condition ε ≤ 0 holds true for disperse systems consisting of dielectric micro particles with ionized donor centers on the surface and free electrons in the bulk. According to Gauss’s theorem, the field strength created in the bulk of a micro particle by the positive surface charge of ionized donor centers vanishes. There are no internal forces that would prevent free electrons from displacement in the bulk of a particle under the action of an external field. This property of the particle is unique. This is not possible for any other material. For this reason, the polarization of micro particles cannot be described within the existing theoretical frame, which asserts that the displacement of electrons under the action exerted by the field forces depends on their interaction with the positively charged centers^8^.

The polarization of powder by an external field results in the displacement of free electrons in the disperse system particles, so that the particles acquire dipole moments oriented in the same direction. The powder polarization density is 10^6^–10^8^ times greater than the polarization density of a uniform dielectric under the same conditions. The unusual electrical properties of powders—spontaneous polarization and amplification of the electric field—are a consequence of their anomalously large polarization density^8^. The polarizing charges located on the opposite surfaces of the powder layer create a constant electric field of the strength **E =  − P**/ε_0_ perpendicular to the faces, where **P** is the system polarization density; ε_0_ is the electrical constant. The value of **E** does not depend on the strength *E*_0_ of the external field^8^:

1 $$- E = P/\varepsilon_{0} = Np/\varepsilon_{0} = Nven_{s} /\varepsilon_{0} ,$$

where *N* is the number of particles per unit volume; *p* = *ven*_s_ is dipole moment of a spherical particle; *v* is particle volume; *e* is electron charge; *n*_s_ is the concentration of ionized donor centers on the particle surface. Using, for example, the values *vN* = 1/2; *n*_s_ = 10^16^ m^−2^, we get: *E* = 10^8^ V/m. The effect of amplifying the electric field by a factor of 10^4^ with zero ohmic losses was observed when the powders were polarized by a sinusoidal electric field. The main property of the polarized powder is expressed by the inequality *E* >  > *E*_0,_ where *E* is the field strength in the powder; *E*_0_ is the strength of the external field that causes the powder polarization. An increase in the field energy ε_0_*E*^2^/2 is due to a decrease in the self-energy of the powder *F*_e_ = (**E**, **P**)/2 =  − ε_0_*E*^2^/2, which corresponds to the value ε = 0^8^.

In the present article, we argue for the vanishing of the powder Ohmic resistance, in which the conduction electrons do not receive energy from the electric field because of a particular state its polarization. The Ohmic loss becomes impossible.

## Results and discussion

Let us determine the mechanism of electrical conductivity of powder consisting of dielectric nanoparticles with ionized donor centers on the surface and free electrons in the bulk. The energy of a powder in the polarized state equals the energy of electrostatic interaction polarization charges + *q*_i_ and − *q*_i_ of the particle dipoles:

2 $$W_{{\text{e}}} = \frac{1}{2}\sum\limits_{i = 1}^{2N} {q_{i} \varphi_{i} } < 0,$$

where φ_i_ is the field potential created at the location of the *i*-th charge by all other charges. There are no internal forces that would prevent free electrons from displacement in the bulk of a particle under the action of an external field. For this reason, after the external field is switched off, the polarization state of the powder with the energy *W*_e_ < 0 remains unchanged. In order to decrease the polarization charges + *q*_i_ and − *q*_i_ of the particles, external forces must do work. In the absence of an external field, the polarized powder is in a stable state with a negative free energy *F.* In this case, the static dielectric permittivity of the powder is negative^8^.

**Figure 1:**
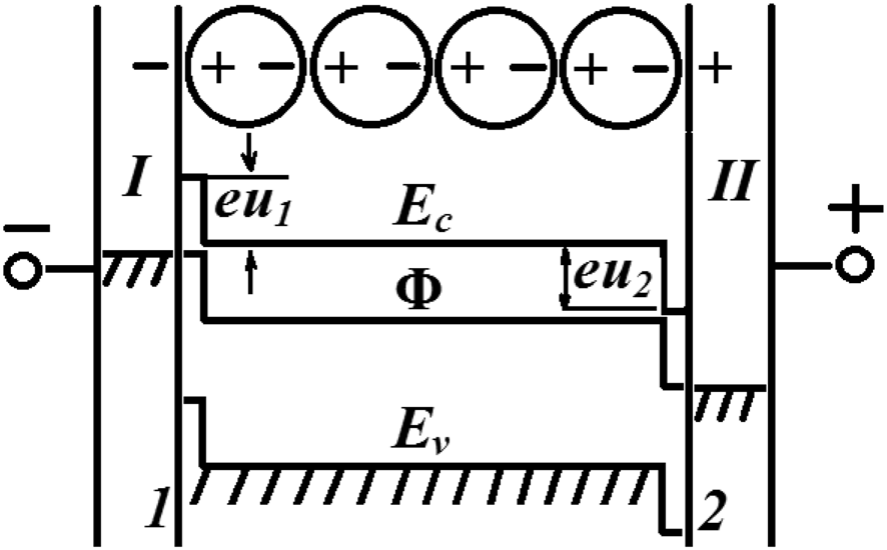
Energy-level diagram of the metal-powder-metal system included to an electrical circuit with a direct current. The powder is conventionally shown as a chain of polarized particles. *E*_c_ is the bottom of the semiconductor conduction band; Φ is the Fermi level; *u*_1_ and *u*_2_ are contact differences of potentials between the polarized powder and electrodes *I* and *II*.

Let a plane-parallel layer of powder be in contact with identical metal electrodes connected to an electrical circuit with a current run by a source of electromotive force (EMF). At distances *L* >  > *l*, where *l* is the particle diameter, the energy-level scheme of the powder coincides with that of an inhomogeneous *n*-type semiconductor^3^. After the electrodes are coupled to the power source, an electric current arises in the circuit and the powder is polarized by the electric field of the EMF source. The powder particles acquire dipole moments. A double electrical layer is formed between the powder surface layer and each of the electrodes (Fig. 1). The powder surface *1* acquires a positive charge + σ and the surface *2* a negative charge − σ. The thermionic work function of the powder surface *1* decreases, while that of the surface *2* increases. Electrons pass from electrode *II* to the powder and from the powder to electrode *I*. There arise contact difference of potentials *u*_1_ and *u*_2_ on the electrode-powder boundaries. A difference of potentials appears between the metal electrodes equal to the sum of their contact differences potentials with the powder: *u*_0_ = *u*_1_ + *u*_2_. Electrode *I* acquires a negative electrical potential and electrode *II* positive.

In the presence of a current, the polarized powder is in a unique state. For a powder included in a closed circuit, the conditions **E =  − P**/ε_0_ and *E* >  > *E*_0_ [Eq. (1)] cannot be satisfied. The powder polarization density is limited by the equality

$$E = E_{0} {-}P/\varepsilon_{0} ,$$

where *E* is the strength of the field in the powder, *E*_0_ is EMF source field strength. This is caused by the fact that the opposite surfaces of the powder layer are connected through circuit elements (electrodes, resistor, source power). The inversion of the field in the powder (*P* > ε_0_*E*_0_, *E* < 0) would correspond to the reversal of the powder current, which is impossible. The current direction in the circuit is set by the EMF source. For allowed values *E* ≥ 0, the condition *E* = 0 corresponds to the maximum polarization density *P*_max_ = ε_0_*E*_0_, the minima of the proper energy *F*_e_ = (**E**, **P**)/2 and the free energy *F* = εε_0_*E*^2^/2 of the polarized powder. The equalities

3 $$E = \, 0; F_{e\min } = \, 0; F_{\min } = \, 0; u_{0} = u_{1} + u_{2}$$

are satisfied independently on the intensity of the circuit current. Local fields of polarized powder particles increase the probability of electron transitions between them and through interfaces *1* and *2*. The scattering of electrons in the powder does not affect the current intensity, since each particle exchanges different electrons with two neighboring particles (hopping conductivity^3^). It follows from *E* = 0 that Ohmic loss (**j**, **E**) and the powder Ohmic resistance *R* = *EL*/*J* are zero, where *L* is the thickness of the powder layer and *J* is the circuit current intensity.

We use Eq. **E**(0, **k**) = **D**(0, **k**)/ε(0, **k**), where **D**(0, **k**) is the field induction. The static permittivity satisfies the equality^2^: 1/ε(0, **k**) = 1–*I*, where

$$I = 2\int\limits_{0}^{\infty} \alpha_{k} (W, {\bf k}) \frac{dW}{W} \geq 0;$$

σ_k_(*W*, **k**) in formula σ_k_ is the quantity associated with the form factor of inelastic electron scattering. The condition of the field *E* attenuation to zero (**D**(0, **k**) ≠ 0, **E**(0, **k**) = 0) corresponds to the equalities *I* = 1 and 1/ε(0, **k**) = 0.

The drift of electrons in the powder under the influence of the field is impossible (*E* = 0). Let us assume that the electric current of density *j* in the powder occurs on account of the electron diffusion^3^:

4 $$j = eD{\text{d}}n/{\text{d}}x = \mu n\left( x \right){\text{d}}\Phi /{\text{d}}x,$$

where *D* is the electron diffusion coefficient; *n* is the electron concentration in a particle; μ is electron mobility in the powder; Φ is the Fermi energy; the axis *x* is directed along the normal to the powder layer. The concentration of electrons in a powder particle depends on the surface density *n*_s_ of ionized donor centers:

5 $$n = {6}n_{{\text{s}}} /l.$$

The gradient of electron density d*n*/d*x* in the powder may arise in two cases. First, the Eq. (5) is satisfied in different particles, but according to the condition d*n*/d*x* ≠ 0, the quantities *n* and *n*_s_ in them depend on the location of the particle in the powder. It means that the particle dipole moments *p* ~ *n*_s_^8^ and the powder polarization state depend on the current density, determined by the gradient d*n*/d*x*, which violates the equalities (3). Second, the condition of electrical neutrality of the powder particles [Eq. (5)] is not satisfied. Then an electric field would arise between the positive and negative bulk charges created by the charged powder particles. This also violates the Eq. (3). Therefore, the condition (4) contradicts the equalities (3).

Since the particles interact through their local micro fields, the states of free electrons of different particles in the polarized powder are self-consistent; i.e., the electrons form a single system. Due to the difference in the electrochemical potentials of the powder and electrodes, electrons pass from electrode *I* to the powder, and from the powder to electrode *II* (see Fig. 1). This causes self-consistent transitions of electrons between the powder particles, owing to which, the polarization charges + *q*_i_ and − *q*_i_, the powder polarization density *P*, and the state of the system of charges *q*_i_ with the energy *W*_e_ (see (2)) are preserved. The cause of the electric current in the powder is the exchange–correlation interaction of electrons on account of the fulfillment of the conditions *E* = 0, *F* = *F*_min_ = 0 for a polarized powder. Surface ionized donor centers increase the electron de Broglie wavelength λ along the semiconductor surface^3^. The electric current in the volume of powder nanoparticles is due to the fact that for electrons in nanoparticles, the value λ ≈ 30–100 nm is commensurate with the particle diameter *l* ≈ 100 nm. Due to the equality *E* = 0, in case of violation of the specified condition, the movement of electrons in the volume of dielectric particles becomes impossible.

**Figure 2:**
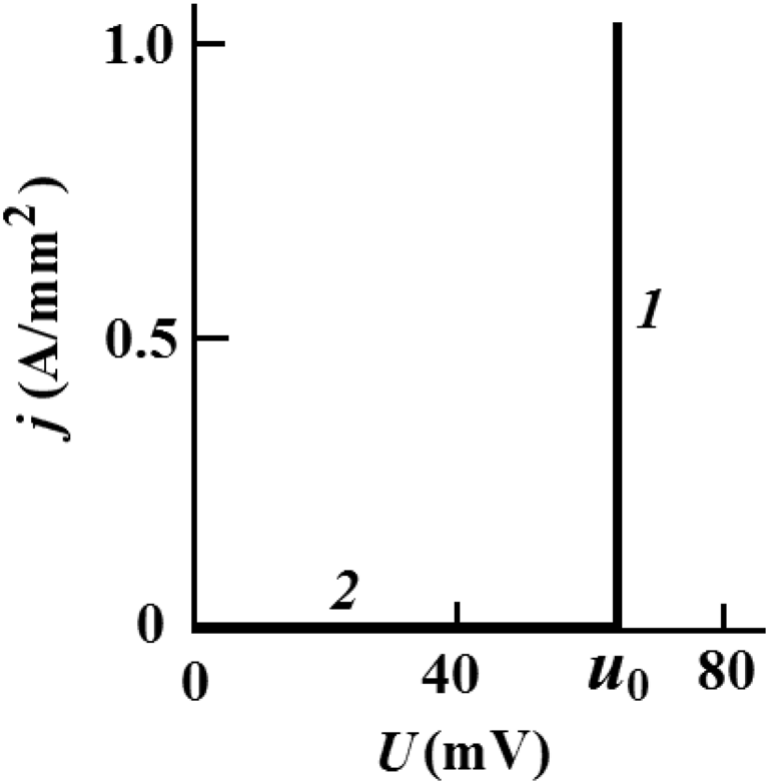
Current—Voltage characteristic of the metal-powder-metal system. *1* before and *2* after change on the electrodes voltage polarity *U*; *j* is the current density; *U* is the voltage between the electrodes; *u*_0_ = *u*_1_ + *u*_2_ is the sum of the contact potential differences of the powder with the electrodes. The powder is in the stable polarized state.

The current–voltage characteristic (CVC) of the metal-powder-metal system *j*(*U*) is a vertical line for the current density *j* ≥ 0. The electrodes voltage does not depend on *j*: *U* = *u*_0_ (Fig. 2, line *1*), which corresponds to the stable state of the powder polarization. In this case, the condition *JR* = 0 is satisfied, where *J* is the current in the powder, *R* = 0 is its ohmic resistance. According to expression (2), after reversing the voltage polarity between the electrodes, the previous powder polarization state and the corresponding difference of electrochemical potentials between the electrodes are preserved. It prevents electrons from moving in the metal-powder-metal system. Local fields of polarized powder particles reduce the probability of electron transitions between them and through interfaces *1* and *2*.The powder current intensity abruptly decreases (Fig. 2, line *2*).

In a ring of nanoparticles, by electromagnetic induction EMF pulse, one can simultaneously create a current and a polarized state of a powder. The state of the polarized ring is unique (*E*_0_ = 0; *W*_e_ ≠ 0; *P* ≅ *Np*_max_ ≅ *Nven*_s_). The current in the polarized ring may be undamped.

## Methods

Materials and measurement methods are described in detail in article^9^. In the experiments, Ni particles with NiO oxide films on their surface and/or Al_2_O_3_ particles were used in the form of balls with an average diameter of 100 nm. The shape, size, and elemental composition of the powder particles were determined using a JSM-6380LV electron microscope with an INCA energy dispersive attachment. A polished silicon crystal with an aluminum film deposited on its surface served as the substrate. The surface of the electrodes was examined using an SMM-2000 atomic force microscope and an Axioscop 2MAT optical microscope. The thickness of the layer of powder particles deposited onto the substrate was *d* ≈ 10 μm. The substrate with the applied powder film was placed into a vacuum chamber made of glass. Hydrogen was continuously pumped through the chamber with a pressure of 10 Pa. The hydrogen purity was 99.995%. Using a high-frequency discharge in a gas (*f* = 40 MHz, *P*_max_ = 30 W), hydrogen plasma was obtained, which from the discharge tube entered the vacuum chamber and interacted with the finely dispersed film. The concentration of free H atoms in the chamber was ~ 10^15^ cm^−3^. The powder layer was kept in plasma at a temperature of 400 K for up to 1.5 h. H atoms on the surface of the dielectrics are in the form of positively charged H^+^ ions, forming shallow donor levels in the band gap of semiconductors^3^. A closed electrical circuit was used, consisting of series connected circuits: a source of stabilized electric voltage, a variable resistor, an ammeter and a layer of nanoparticles between the electrodes. The dependence of *J*(*U*) was measured, where *J* is the direct current in the circuit and *U* is the electrical voltage between the electrodes in contact with the powder. The powder was in the atmosphere of hydrogen (Ni) or air (Al_2_O_3_) at a temperature of 295 K. In the case of the direct contact of two electrodes (without a layer of powder between them), the CVC was linear in accordance with Ohm’s law.

In the constant electric current in Al_2_O_3_ and NiO powders, containing donor centers in the form of interstitial hydrogen atoms on the surface of its spherical nanoparticles, Ohmic resistance is zero. The dependence *j*(*U*) shown in Fig. 2 (lines *1* and *2*) coincides with the experimentally observed dependences^9^.

The experimentally obtained CVC *J*(*U*) for powders (vertical lines at an inter-electrode voltage *U* = *u*_0_) cannot be an analogue of electrical breakdown and avalanche increase in the reverse-bias region of the silicon diode (Zener diode). The maximum kinetic energy of free electrons in the powder, *eu*_0_, is tens of times less than the dielectric band gap ∆*E*. This makes ionization powders by electron impact impossible. In the case of powders of nickel nanoparticles or of aluminum oxide, the voltage between the electrodes *u*_0_ is respectively 0.035 and 0.2 V^9^. For nickel oxide films on the surface of nanoparticles nickel ∆*E*_1_ = 3.5–4.0 eV; for thin films of aluminum oxide ∆*E*_2_ = 4.3–4.6 eV^10^.

The presence of ionized donor states on the surface and free electrons in the bulk of nanoparticles of powder dielectrics follows from two facts. The original powders are not electrically conductive. Their conductivity arises after their exposition in the atomic hydrogen medium. CVC of the film has the form of a vertical line, which is a consequence of arising ionized donor centers on the particle surface. After a long exposition of powders in the atomic hydrogen, CVC of the film corresponds to Ohm’s law^9^. It means that hydrogen atoms penetrate from the surface into the bulk of nanoparticles.

Additional experiments have also been carried out. Dielectric hysteresis loops were measured for layers of arsenic-doped (*n*_As_ = 10^17^ cm^−3^) fine-dispersed germanium (average size of micro particles was 16 µm) at a temperature of 295–540 К. The loops have the shape of ellipses with a positive slant with a temperature dependent area. No effects of anomalously high powder polarization density and zero ohmic loss have been detected. It follows from this fact that the unusual electrical properties of the powders are caused by the location of the donor centers on the surface of the particles^8^.

## Conclusion

We have substantiated the structure of a solid (powder), owing to which the Ohmic resistance is zero. The electrical field of the current source brings about the transition of the powder into a polarized stated with the minimal possible energy. The local fields of the polarized particles increase the probability of electron transition between the particles in one direction and decrease the transition probability in the opposite direction. The electrochemical potentials of the opposite surfaces of the polarized powder layer are different. For these reasons, a constant electrical current is possible in its bulk at the zero strength of the electric field. The electric current in the powder without energy loss occurs due to the self-consistent movement of electrons between the polarized particles of the powder. The mechanism of electrical conductivity in powders is similar to the hopping conductivity in a uniform semiconductor. One of the particle free electrons moves between the powder particles. The theoretical results and experimental observations are found in agreement.

According to the obtained results and the results of the work^8^, the powder layer with donor centers on the surface of the powder particles, depending on the values of *J* and *E*_0_, has the following characteristics:

6 $$J \ne 0,E_{0} \ne 0; E \, = 0,P \ne 0,{1}/\varepsilon = \, 0,F_{{\text{e}}} = \, 0,F_{{}} = \, 0;$$

7 $$J = \, 0,E_{0} \ne 0; |E| > > E_{0} ,P \ne 0,\varepsilon \, = \, 0,F_{{\text{e}}} < 0,F_{{}} = \, 0;$$

8 $$J = \, 0,E_{0} = 0; E \ne 0,P \ne 0,\varepsilon \, < \, 0,F_{{\text{e}}} < 0,F < 0,$$

where *J* is the current strength in the powder; *E*_0_ is the strength of the external field; *E* is the strength of the field in volume of the powder; *P* is the system polarization density; ε is the static permittivity of the powder; *F*_e_ = (**E**, **P**)/2; *F* = εε_0_*E*^2^/2.

## Data Availability

All data needed to evaluate the conclusions of this study are available in the main text. The data that support the findings of this study are available from the corresponding author upon reasonable request.
